# PKM2 inhibition may reverse therapeutic resistance to transarterial chemoembolization in hepatocellular carcinoma

**DOI:** 10.1186/s13046-020-01605-y

**Published:** 2020-06-03

**Authors:** Sean P. Martin, Valerie Fako, Hien Dang, Dana A. Dominguez, Subreen Khatib, Lichun Ma, Haiyang Wang, Wei Zheng, Xin Wei Wang

**Affiliations:** 1 Laboratory of Human Carcinogenesis and Liver Cancer Program, Center for Cancer Research, National Cancer Institute, NIH, 37 Convent Drive, MSC 4258, Building 37, Room 3044A, Bethesda, MD 20892 USA; 2 Department of Surgery, Division of Surgical Research, Thomas Jefferson University, Philadelphia, PA USA; 3 Division of Pre-Clinical Innovation, Therapeutics for Rare and Neglected Diseases, National Center for Advancing Translational Sciences, Rockville, MD 20850 USA; 4 Liver Cancer Program, Center for Cancer Research, National Cancer Institute, Bethesda, MD 20892 USA

**Keywords:** Hepatocellular carcinoma, TACE, Therapeutic reprogramming, PKM2, Shikonin

## Abstract

**Background:**

Therapeutic options for patients with hepatocellular carcinoma (HCC) are limited. Transarterial chemoembolization (TACE) is an interventional procedure used to deliver chemotherapy and embolizing agents directly to the tumor and is the procedure of choice for patients with intermediate stage HCC. While effective, more than 40% of patients do not respond to therapy, highlighting the need to investigate possible mechanisms of resistance. We sought to evaluate mechanisms of TACE resistance and evaluate a potential therapeutic target to overcome this resistance.

**Methods:**

Using a prognostic gene signature which predicts TACE response (TACE Navigator) in a cohort of HCC patients who received TACE, patients were classified as responders and non-responders. Transcriptomic and gene pathway analysis were used to identify potential drivers of TACE resistance. Knockdown of the gene encoding rate limiting enzyme PKM2 using shRNA in HCC cell lines, as well as pharmacologic inhibition of PKM2 with shikonin using an in vitro TACE model measured response to chemotherapy under hypoxia. Finally, we replicated the TACE model with shikonin using patient derived cell line organoids (PDC). Functional studies were performed in vitro using immunoblotting, quantitative polymerase chain reaction, glycolysis and hypoxia assays.

**Results:**

In patient non-responders, we identified enrichment of the glycolysis pathway, specifically of the gene encoding the rate-limiting enzyme PKM2. We identified four HCC cell lines which recapitulated a TACE responder-like and non-responder-like phenotype. PKM2 knockdown in HCC cell lines demonstrated a less proliferative and aggressive phenotype as well as improved drug sensitivity to both doxorubicin and cisplatin. In vitro TACE model demonstrated that TACE non-responder-like cells overcame therapeutic resistance and rendered them susceptible to therapy through PKM2 knockdown. Lastly, we obtained similar results using a pharmacologic PKM2 inhibitor, shikonin in both cell lines, and PDC organoids.

**Conclusion:**

Elevated PKM2 is associated with treatment resistance and abbreviated survival in patients receiving TACE. Elevated PKM2 in vitro is associated with increased utilization of the glycolysis pathway, resulting in oxygen independent cell metabolism. Through PKM2 knockdown as well as with pharmacologic inhibition with shikonin, non-responder cells can be reprogrammed to act as responders and could improve TACE efficacy in patients.

## Background

Hepatocellular carcinoma (HCC) is the most common primary tumor of the liver and with approximately 850,000 new cases per year, represents the fourth leading cause of cancer related death globally [1, 2]. In the United States, liver cancer is the fastest rising malignancy and while the incidence pales in comparison to other malignancies, it is among the top causes of cancer related death [3]. Despite advances in identifying the etiology of disease, advances in durable curative therapy has not been as fruitful. One area of promise is in the treatment of intermediate stage HCC (Barcelona Clinic Liver Cancer Stage B) with transarterial chemoembolization (TACE). TACE is a procedure in which a catheter is directed into the feeding arterial branches of the tumor and chemotherapy, commonly doxorubicin, is directly infused into the tumor, followed by embolization of the feeding artery [4]. With treatment related mortality less than 5%, TACE can be expected to result in a median survival of 11–20 months [5, 6]. While this procedure is life prolonging for many, more than 40% of patient have no objective response to treatment and are left with far less effective systemic therapies [7].

Due to the large number of patients that do not respond to TACE, it is imperative to identify the drivers that promote resistance. Recently, we developed a gene signature predictive of HCC patient response to TACE treatment [8]. We showed hypoxia signaling and glycolysis/gluconeogenesis-related pathways were activated in HCC patients that did not respond to TACE. Accordingly, we hypothesized that preferential utilization of the glycolysis pathway allows HCC to evade TACE. In the present study, we demonstrate that TACE resistance is associated with alterations in glucose metabolism, specifically through the enrichment of the glycolysis related gene, Pyruvate kinase muscle isozyme M2 (PKM2). PKM2 is a splice variant of the of pyruvate kinase and its role in cancer cell metabolism has been associated with propagation of the Warburg effect, allowing for a selective growth advantage of malignant cells [9]. Using transcriptomic data from HCC patients, we determined that TACE non-responders have elevated PKM2 expression and demonstrated that PKM2 inhibition via gene silencing or a pharmacological agent shikonin sensitized HCC cell lines and patient-derived cell lines (PDCs) to chemotherapeutic treatment in a hypoxic environment mimicking the TACE procedure. We believe that these findings may lead to a novel treatment adjunct to TACE, warranting further clinical testing.

## Methods

### Patient and cell line cohorts

The Liver Cancer Institute (LCI) cohort is a publicly available HCC dataset available at the Gene Expression Omnibus (GEO, GSE14520; http://www.ncbi.nlm.nih.gov/geo). Data from this cohort has been previously described [10]. For this study, only the 105 patients who underwent TACE were selected from the LCI cohort. The Hong Kong cohort consists of 47 patients who underwent TACE, and has previously been described in detail [8]. Patients from these cohorts were assigned as TACE responders based on the previously described TACE Navigator gene signature utilizing NanoString technology (Seattle, Washington) [8]. NanoString was also utilized to evaluate for PKM2 expression in the Hong Kong cohort and PDCs. Gene expression as measured by NanoString counts, were log_2_ transformed and converted to z-score within each cohort.

HCC cell line transcriptomic data was downloaded from the Cancer Cell Line Encyclopedia (CCLE) and log_2_ transformed [11]. Expression data was then z-score transformed and the TACE Navigator prognostic signature was then applied. Based on this data, cell lines were classified as responder and non-responder like and used for downstream analysis.

### Cell lines and plasmid

HUH7 were cultured in Dulbecco’s modified Eagles Medium (Life Technologies) supplemented with 10% fetal bovine serum (FBS), penicillin, streptomycin and L-glutamine. Hep3B were cultured in Minimal Essential Medium (Life Technologies) supplemented with 10% FBS, penicillin, streptomycin, L-glutamine, non-essential amino acids and sodium pyruvate. SNU-387 were cultured in RPMI-1640 (Life Technologies) supplemented with 10% FBS, penicillin and streptomycin. SNU-475 were cultured in RPMI-1640 supplemented with 10% heat inactivated FBS, penicillin and streptomycin. PDCs (HCC3501, HCC 3796, HCC 4006, and HCC3258) were cultured in Dulbecco’s modified Eagles Medium: Nutrient Mixture F-12 (Life Technologies) supplemented with 10% FBS, non-essential amino acids, penicillin and streptomycin. pGFP-C-shLenti-PKM2 shRNA was purchased from Origene and packaged using FuGENE 6 transfection reagent (Promega). Cells lines were passaged less than 15X after thaw.

### Immuno-blotting and quantitative RT-PCR

Protein lysates were separated on Bis-tris 4–12% gels (Life Technologies) and transferred to a nitrocellulose membrane (Life Technologies). Protein detection was performed using anti-PKM2 (Cell Signaling Technology, cat# 4053S) and anti-β-actin (Sigma-Aldrich, cat# A5316). All immunoblots are cropped for viewing. Total RNA was extracted using Trizol (Life Technologies) according to the manufacturer’s protocol. cDNA was synthesized using High Capacity cDNA Reverse Transcription Kit (Life Technologies). Gene expression was quantified by quantitative real-time transcription-polymerase chain reaction (qRT-PCR) using CFX384 Real Time System (BioRad) and SYBR Green PCR Master Mix (GenePharma). Gene expression levels were normalized to β-actin. The relative gene expression levels were detected and calculated using the ΔΔCt comparative method. The RT-PCR primer sequences are as follows: PKM2, 5′- ACTGTCCTCACCAAGTCTGG-3′ (forward) 5′-GAAGATGCCACGGTACAGGT-3′ (reverse); β-Actin, 5′-TTGTTACAGGAAGTCCCTTGCC-3′ (forward) 5′-ATGCTATCACCTCCCCTGTGT-3′ (reverse).

### Glycolysis assay

Glycolysis was measured using a commercially available kit (Cayman Chemical, cat#600450). Briefly, cells were seeded at 20,000 cells/well in triplicate in a 96-well plate in FBS free media appropriate for each cell line. Cells were placed in 37 °C incubator for 48 h. For hypoxia induced measurements cells were placed in a 37 °C incubator for 48 h in a closed 1% oxygen environment. After 48 h, plates were centrifuged at 1000 rpm for 5 min and 10 μl of supernatant was removed from each well and added to the reaction solution. The plate was incubated with shaking for 30 min and the absorbance at 490 nm was measured.

### Cell Colony formation

Cells were seeded at 500 cells/well in a 6 well plate and cultured for 10 days in both normoxia and 1% oxygen in parallel. Cells were then washed with ice cold 1x PBS (Life Technologies) followed by ice cold fixation with methanol for 30 min. After fixation, cells were washed with dH_2_O followed by 2 h of 0.05% crystal violet staining and subsequent washings with dH_2_O. Colonies were quantified via manual counting in triplicates.

### Cell proliferation and migration/invasion assays

Xcelligence (ACEA Bioscience) assays were performed for cell proliferation, cell invasion and cell migration and measured by cell index. Cell index is a measure of cell proliferation within each well of the plate as defined by (impedance at time point – impedance without cells) / nominal impedance value. Assays were performed in closed 1% hypoxic environment. Cells were seeded at 1000 cells/well in E-plates (ACEA Bioscience, cat # 5232368001), with appropriate medium in quadruplets. For TACE simulation assay, appropriate wells were treated with doxorubicin (Sigma, cat# D1515) and/or shikonin, (Sigma, cat#S7576) at the IC_50_ appropriate for each cell line (Supplemental Table 1). Cell specific IC_50_ was determined based on data from the Genomics of Drug Sensitivity in Cancer database [12]. Proliferation was measured over 72 h. For migration and invasion assay, 30,000 cells/well were plated on a CIM plate (ACEA Bioscience, cat #5665817001) with Matrigel (for invasion only) in a 1:200 dilution with appropriate media. Migration and invasion were measured over 24 h. For cell proliferation, migration and invasion experiments were performed in triplicate.

### Apoptosis assay

Relative apoptosis activity was measured via Caspase-3/7 activity by using the Apo-ONE homogeneous Caspase 3/7 assay (Promega, cat#G7792). Briefly cells were seeded in cell specific media at 7500 cells/well in a 96 well plate and placed in a 37 °C incubator overnight. Cells were then placed in a hypoxic incubator for 48 h. The assay was then performed per the manufacturer’s protocol and fluorescence was measured (excitation: 485 nm, emission: 535 nm).

### Cell viability and drug sensitivity

Cells were seeded in cell specific media at 7500 cells/well in a white 96 well plate and placed in a 37 °C incubator for 24 h. For doxorubicin-only experiments, two-fold drug dilutions were performed. For doxorubicin and shikonin experiments, a similar two-fold doxorubicin dilution was performed. Following dilutions, shikonin was added at the cell specific IC_50_. One plate was placed in a normal 37 °C incubator and the other plate was placed in hypoxia. Normoxia/hypoxia experiments were performed concurrently. After 48-h incubation, plates were removed from the incubator and allowed to reach room temperature (RT). At this point Cell-Titer Glo (Promega) was added to each well. Plates were placed on an orbital shaker for 2 min and then incubated for 10 min at RT. Luminescence was then measured. Viability assays are relative to the normoxia untreated control. For combination index experiments, a shikonin two-fold dilution was performed in addition to the previously described. To calculate the combination index, a constant ratio of 1:1 doxorubicin and shikonin was used. Viability and drug sensitivity assays were also performed with cisplatin (Milipore Sigma, cat# 232120) in the same manner. Combination index constant ratio was performed at a ratio of 4:1 cisplatin to shikonin given the relatively high IC_50_ of cisplatin in HCC cell lines.

### Organotypic culture and viability

PDCs were cultured in AlgiMatrix 3D Culture System (Life Technologies) in the method described by Takai et al. [13]. Briefly, one million cells were seeded and cultured in cell specific media for 2 weeks at which point they were treated with doxorubicin or shikonin alone as well as in combination and placed in hypoxia for 48 h. Haematoxylin and eosin staining was performed before and after treatment. For the collection of spheroids, the matrix was dissolved using Algimatrix Dissolving Buffer (Life Technologies) per the manufacturer’s instructions. Organoids were then pelleted by centrifugation at 300 g for 5 min. After collection, organoids were seeded in a white 96 cell plate with the addition of Cell Titer Glo 3D Cell Viability Assay (Promega, cat# G9683). Plates were placed on an orbital shaker and allowed to incubate per the manufacturer’s instructions. Luminescence was then measured, and viability was calculated relative to the untreated control.

### Statistical analysis

For all statistical analyses, a *p* < 0.05 was considered statistically significant. Statistical tests included 2-tailed Student’s t-test for two groups or one-way ANOVA for multiple groups where appropriate. For clinical data, descriptive statistics were calculated for all variables of interest. Continuous variables were summarized using means, whereas categorical variables were summarized using frequency and percentages. Comparisons of categorical variables were performed using the chi-square or Fisher’s exact test whereas continuous variables were compared with the two-sided Student’s t-test. Overall survival (OS) was calculated based on the provided survival times utilizing the Kaplain-Meier method and log-rank test. Following a univariable analysis, a Cox proportional hazards model was constructed using all variables with *p* < 0.05. Genomic analyses were performed using BRB-ArrayTools version 4.6.0 (Bethesda, MD). Experimental statistics were performed using GraphPad Prism 7.01 (San Diego, CA). Clinical statistics were performed using STATA 14.0 (College Station, TX). Combination index was calculated using the Chou-Talalay method and Compusyn software [14, 15].

## Results

### Alterations in glucose metabolism are correlated with TACE response

**Fig. 1:**
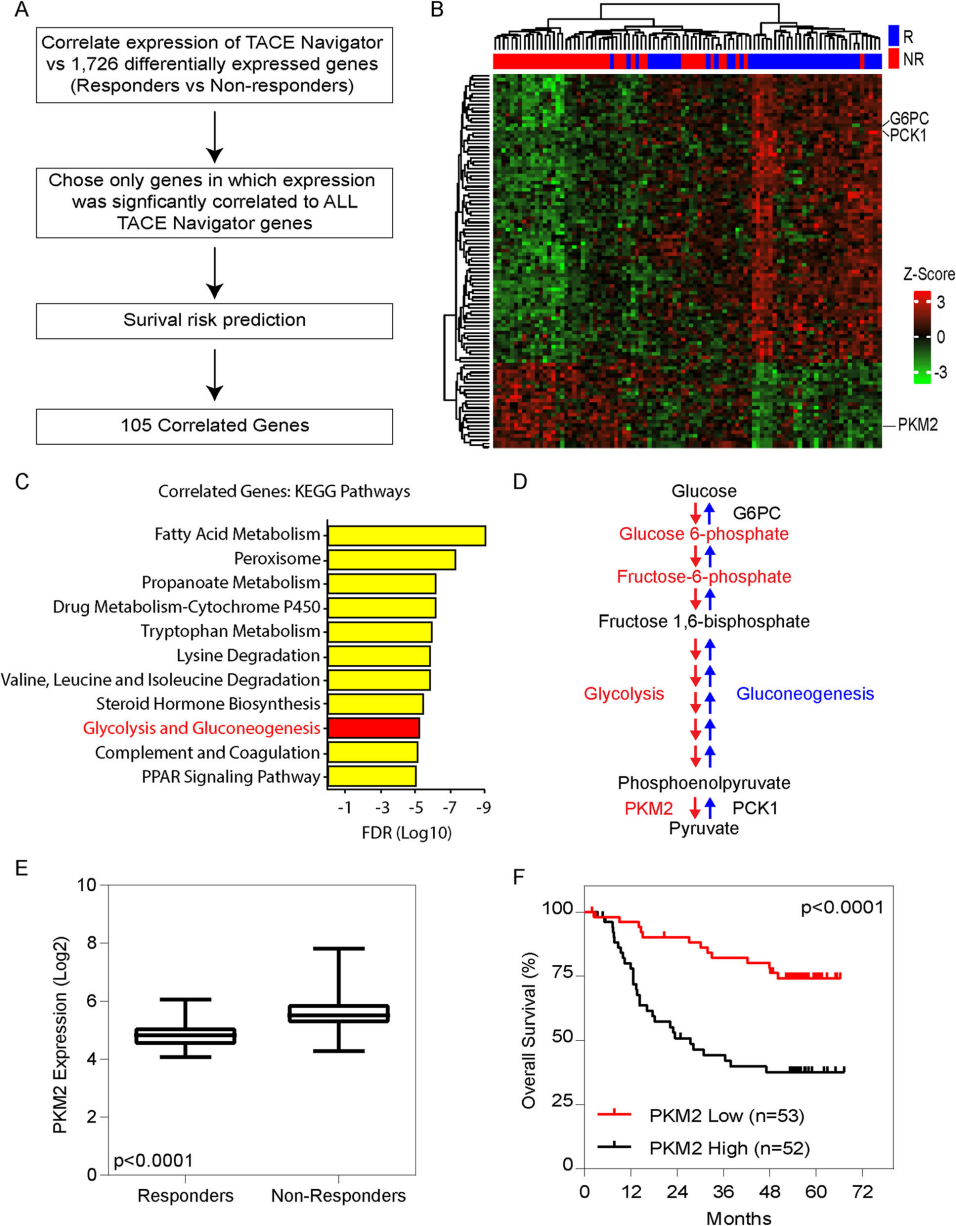
TACE resistance is associated with alteration in glucose metabolism specifically through the enrichment of PKM2: **(a)** Schematic outline of the identification of 105 genes differentially expressed genes between TACE responders and non-responders which also correlate with the TACE Navigator gene signature. **(b)** Heatmap demonstrating the clustering of TACE responders and non-responders based on the 105 correlated genes. **(c)** KEGG pathway gene set enrichment analysis of 105 correlated survival related genes demonstrating alterations in glucose metabolism as a potential pathway associated with TACE resistance. **(d)** Schematic representation of the glycolysis and gluconeogenesis pathways highlighting the genes G6PC, PCK1 and PKM2, all of which correlate with the TACE Navigator signature. **e** Relative expression of PKM2 between TACE responders and non-responders with associated survival analysis

We first set out to identify signaling pathways associated with TACE resistance by utilizing the TACE Navigator gene signature, which includes 14 genes (ASNS, CDK1, DNASE1L3, FBXL5, GOT2, GRHPR, IARS, LGALS3, LHFPL2, MFGE8, MKI67, PEBP1, TNFSF10 and UBB). The TACE navigator was used to identify two distinct subpopulations, TACE responders and non-responders. Gene expression analyses between the responders and non-responders resulted in 1726 differentially expressed genes, as described previously [8]. To identify genes that are important drivers of resistance and functionally linked to TACE, we correlated the gene expression of the 15 genes from the TACE Navigator to the 1726 differentially expressed genes using Spearman Rank. We reasoned that only genes that are functionally important for TACE resistance will be correlated with all 15 genes. We filtered out genes that were correlated with survival and correlated with all 14 genes from the TACE navigator, resulting in 105 genes (FDR < 0.05) (Fig. 1a). We then performed hierarchical clustering of TACE patients based on our 105 correlated genes and observed two distinct populations (Fig. 1b). We next performed KEGG pathway gene set enrichment analyses to identify unique signaling pathways associated with TACE response. We found correlation with 11 unique KEGG pathways, including glycolysis and gluconeogenesis (Fig. 1c). Given our previous findings that TACE resistance is associated with hypoxic reprograming [8], we investigated the glycolysis and gluconeogenesis pathway, which includes PKM2, Glucose-6-Phosphatase Catalytic Subunit (G6PC) and Phosphoenolpyruvate Carboxykinase 1 (PCK1) (Fig. 1d). These genes were among the most differentially expressed between responders and non-responders (Fig. 1b) and we chose to further investigate PKM2 due to its known role in hypoxia and its interaction with the hypoxia regulatory gene HIF-1α [16].

We next set out to examine the functional significance of each of these genes by examining their relative gene expression in responders and non-responders and whether their expression correlated with overall patient survival. We found that in a cohort of patients that received TACE, PKM2 was elevated in non-responders compared to responders (Fig. 1e; log fold change 1.14, *p* < 0.01). PKM2 expression using a median cutoff was also associated with significantly attenuated overall survival in this patient cohort (Fig. 1f; median survival High: 27.5 months vs Undefined, *p* < 0.0001). Conversely, we found that gluconeogenesis related genes, G6PC and PCK1, had significantly higher expression amongst responders, which was associated with better overall survival (PCK1: median survival High: Undefined, Low: 47.1 months, *p* = 0.05; G6PC: High: Undefined, Low: 47.1 months, *p* = 0.04) (Supplementary Figure 1). These findings indicate that PKM2 is more likely associated with non-responders and the glycolysis pathway. To ensure that these findings were not specific to a single subset of HCC patients, we set out to validate our findings in the Hong Kong cohort (*n* = 47). From this analysis, we found PKM2 was significantly elevated in non-responders and associated with a worse overall survival (High: 27.8 months, Low: Undefined, *p* = 0.05) (Supplementary Figure 2), consistent with the LCI cohort.

Finally, to establish the influence of PKM2 on survival amongst other known prognostic clinical variables, we constructed a Cox Proportional-Hazards Model (Supplemental Table 2). In the univariable analysis, elevated PKM2, BCLC stage B and C, local invasion and cirrhosis were all found to impact survival, which were included in the multivariable model. Multivariable analysis revealed high PKM2 expression was associated with a three-fold increased risk of death (HR 3.02, 95% CI: 1.50–6.04, *p* = 0.002). In addition, BCLC stage C was also found to be significantly associated with death but to a lesser extent (HR 2.62, 95% CI 1.30–5.27, *p* = 0.007). Given these findings, we hypothesized that PKM2 was associated with TACE resistance and warranted further investigation.

### PKM2 and glycolysis in non-responders

**Fig. 2:**
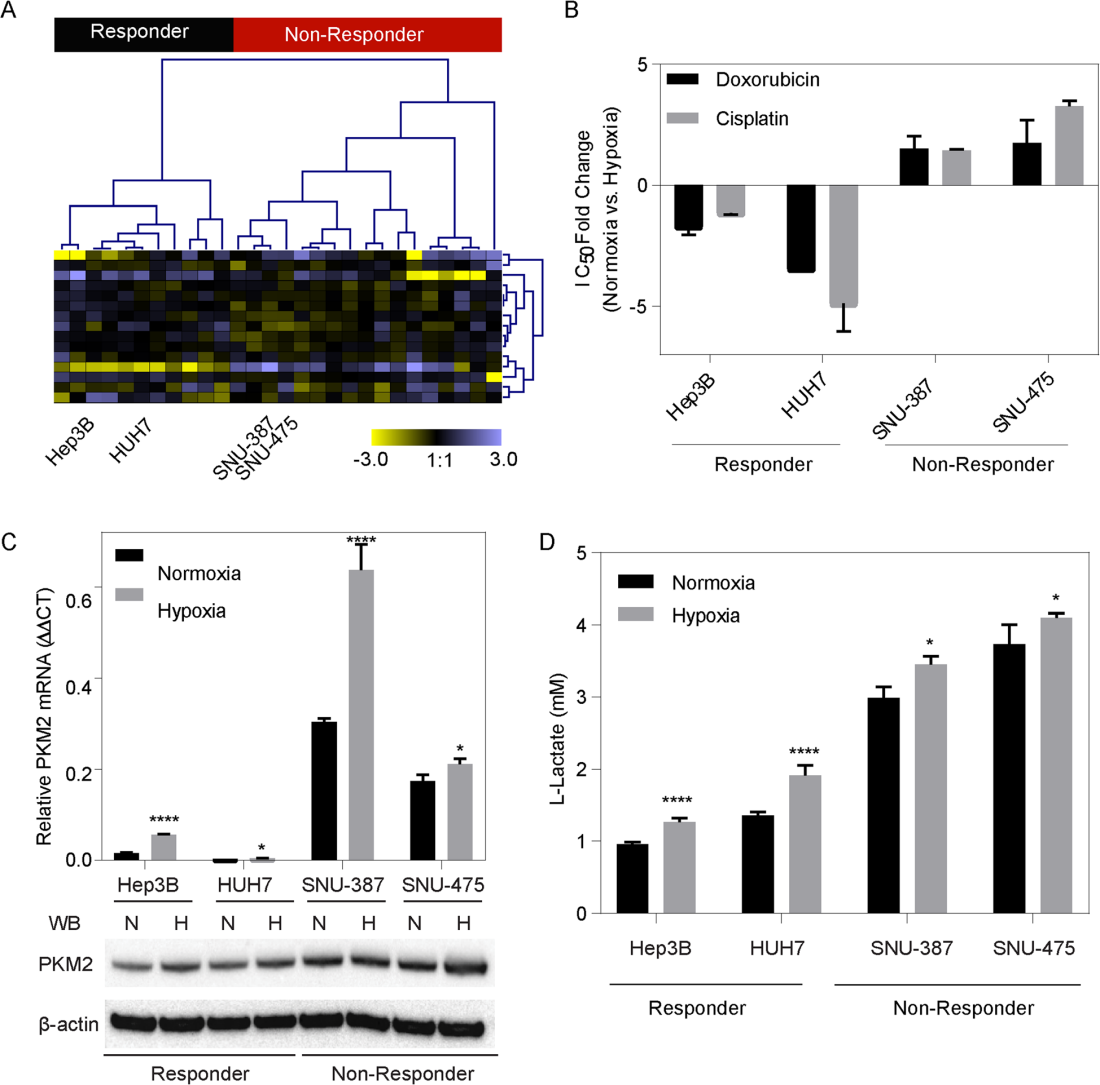
HCC cell lines can serve as a model of TACE resistance in vitro: **(a)** Hierarchical clustering of liver cancer cell lines based on the TACE Navigator gene signature to identify potential responder like and non-responder like cell lines. **(b)** In vitro confirmation of HCC cell line responder and non-responder like phenotypes. Responder like cells have a decrease in drug IC_50_ when subjected to hypoxia whereas non-responder like cells undergo an increase in IC_50_ when subjected to hypoxia **(c)** qPCR demonstrates that TACE non-responder like cells have enrichment of PKM2 mRNA in both normoxia and hypoxia with immuno-blot representing protein expression of PKM2 among responders and non-responders in normoxia (N) and hypoxia (H) demonstrates enrichment of PKM2 in non-responders like cells. **(d)** Glycolysis activity via lactate production of responders and non-responders in both normoxia and hypoxia demonstrates increased glycolytic activity among non-responder like cells

To investigate TACE resistance, we first determined HCC cell lines with the similar transcriptome profiles that we observed within our patient cohorts using the 27 HCC cell lines. Using the 14 TACE Navigator genes, we performed hierarchical clustering analyses, which yielded two groups defined as responder and non-responder based on the expression patterns resembling of HCC patients (Fig. 2a). We then selected two responder-like cell lines, Hep3B and HUH7, as well as two non-responder-like cell lines, SNU-387 and SNU-475 for downstream functional studies as these lines were available in our cell repository. To confirm the responder-like and non-responder-like phenotype, we tested the IC_50_ of the most commonly used TACE chemotherapeutics, doxorubicin and cisplatin, in both normoxia and hypoxia, a condition mimicking the TACE procedure. We observed that in the responder-like cell lines, there was a decrease in the IC_50_ of doxorubicin (Fold change: Hep3B: -1.7, HUH7: − 3.45) and cisplatin (Fold change: Hep3B -1.14, HUH7–4.89) whereas in the non-responder-like cell lines, the IC_50_ increased for both doxorubicin (Fold change: SNU-387: 1.58, SNU-475: 1.74) and cisplatin (Fold change: SNU-387: 1.45, SNU-475: 3.27) when subjected to hypoxia (Fig. 2b). We next characterized the cell lines by determining relative PKM2 mRNA and protein expression under both normoxic and hypoxic conditions. Accordingly, the non-responder-like cell lines had higher PKM2 mRNA and protein expression in normoxia compared to responders, which was further enhanced under hypoxic conditions (Fig. 2c). To assess the functional role of PKM2 expression in glucose metabolism, glycolysis activity was assessed. The non-responder like cells have a higher baseline glycolysis activity as well as enhanced activity in hypoxia (Fig. 2d). Together, these findings are indicative of a difference in glycolysis utilization between responder and non-responder like cell lines.

### PKM2 knockdown results in a less aggressive phenotype

**Fig. 3:**
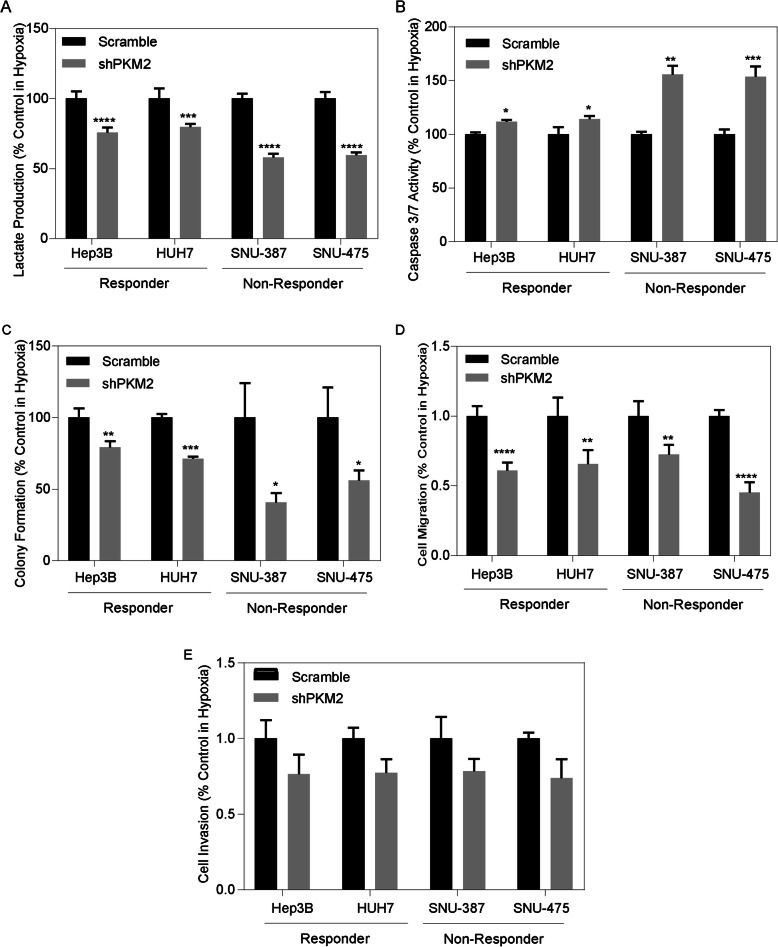
PKM2 Knockdown Affects Cancer Associated Phenotypes: **(a)** shPKM2 results in significant reduction in glycolysis activity in hypoxia among both TACE responder and non-responder like cell lines. **(b)** Significant increase in caspase 3/7 activity in hypoxia with shPKM2 in hypoxia. **(c)** shPKM2 significantly reduced colony formation ability in both responders and non-responders in hypoxia. **(d)** shPKM2 results in significant reduction in migratory ability in hypoxia but has no effect on invasion ability **(e)**

We next investigated the functional role of PKM2 in both responder- and non-responder-like HCC cell lines. We measured lactate production in HCC cells treated with shPKM2 lentivirus and scramble control. PKM2 knockdown resulted in a significant decrease in lactate production across all cell lines (Fig. 3a). Among responder-like cells, Hep3B demonstrated a 24.2% reduction (*p* < 0.01) while HUH7 demonstrated a similar 20.3% reduction (*p* < 0.01). Conversely, non-responder-like cells experienced a greater lactate reduction (42.0% in SNU-387, 40.4% in SNU-475, *p* < 0.01). Apoptosis activity was also increased in all cell lines with a greater effect in the non-responder-like cells (% increase: SNU-387: 55.6%, *p* < 0.01; SNU-475: 53.5%, *p* < 0.01) than in responder-like cells (% increase: Hep3B: 11.7%, *p* = 0.02; HUH7: 14.0%, *p* = 0.02) (Fig. 3b). To test the effect of decreased glycolysis activity on cellular viability and metastatic potential, we performed colony formation, migration and invasion assays. Cell viability, as measured by colony formation, demonstrated that all cell lines were affected, with non-responder-like cells illustrating a greater effect (% decrease: SNU-387: 59.1% *p* = 0.05; SNU-475: 43.9%, *p* = 0.02) than what was observed in responder-like cells (% decrease: Hep3B: 20.8%, *p* < 0.01; HUH7: 20.8%, *p* < 0.01) (Fig. 3c). Cell migration was significantly decreased amongst all cell lines with PKM2 knockdown (Fold change: Hep3B 0.61, *p* < 0.01; HUH7: 0.66, *p* = 0.02; SNU-387: 0.73, *p* = 0.03; SNU-475: 0.45, *p* < 0.01) (Fig. 3d), whereas a reduction in PKM2 had no significant effect on invasion (Fold change: Hep3B: 0.76, *p* = 0.20; HUH7: 0.77, *p* = 0.06; SNU-387: 0.78, *p* = 0.07; SNU-475: 0.74, *p* = 0.10) (Fig. 3e). These data indicate that PKM2 knockdown disrupts glycolytic activity, thus affecting cancer-associated phenotypes. In addition, these data suggest that inhibition of PKM2 may be a useful step to overcome TACE resistance.

### PKM2 knockdown sensitizes non-responder cells to doxorubicin and improves TACE-like response

**Fig. 4:**
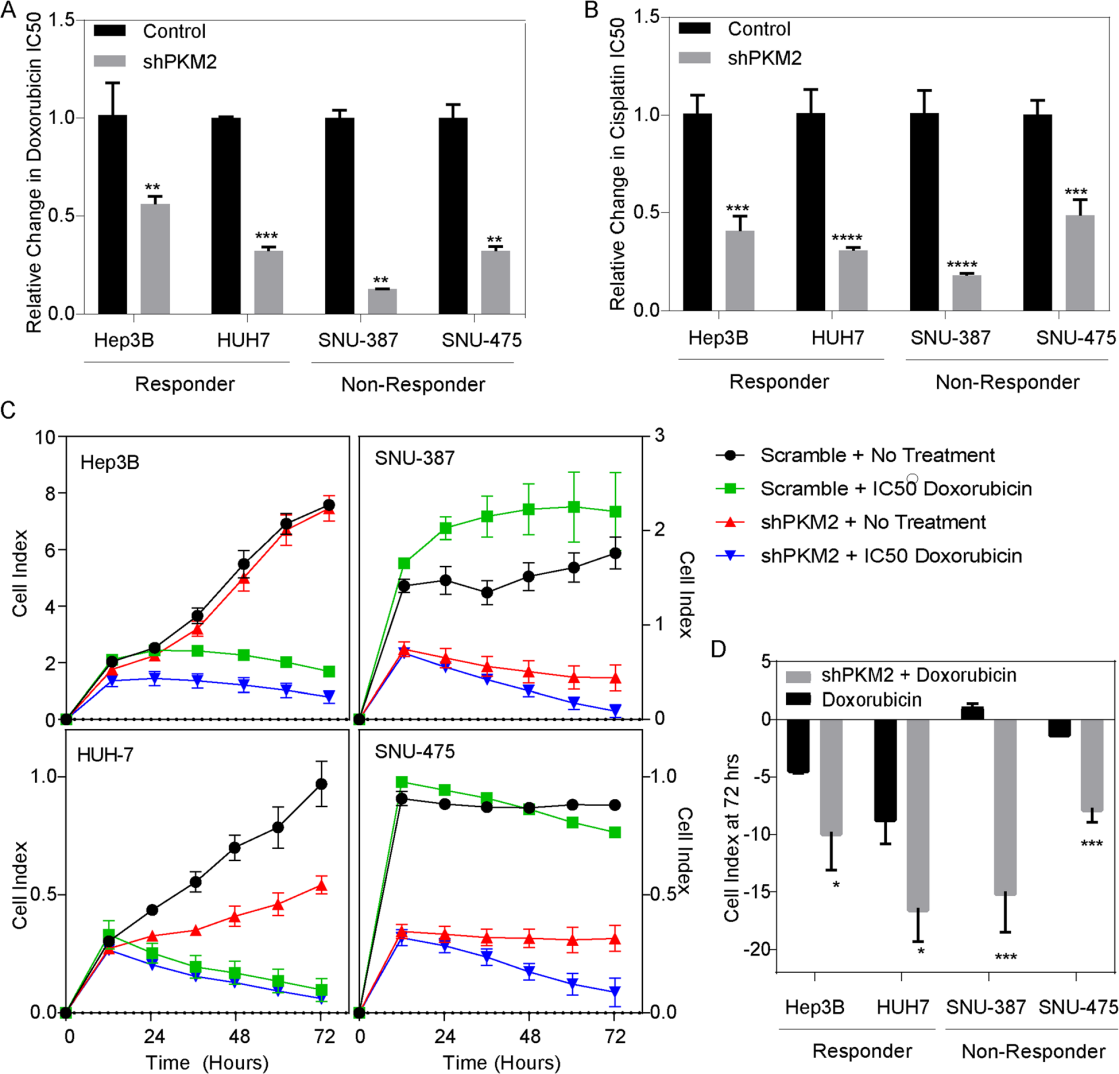
PKM2 Knockdown Sensitizes Cells to Chemotherapy and Improves TACE Response In Vitro: **(a)** PKM2 knockdown results in a significant decrease in the IC_50_ of doxorubicin in hypoxia in both responder and non-responder like cell lines **(b)** PKM2 knockdown results in a significant decrease in the IC_50_ of cisplatin in hypoxia in both responder and non-responder like cell lines **(c)** In vitro TACE assay measuring cellular proliferation at 72 h in hypoxia after the administration of doxorubicin at cell specific IC_50_. TACE responder like cells (left) have a significant reduction in cellular proliferation when treated with doxorubicin alone unlike TACE non-responder like cells (right) which have little to no response. TACE non-responder like cells overcome doxorubicin resistance in hypoxia with the addition of a PKM2 knockdown **(d)** Relative change in cellular proliferation demonstrating improvement of TACE efficacy in responder like cells and therapeutic reprogramming in TACE non-responders with shPKM2

We hypothesized that PKM2 knockdown could improve TACE efficacy. We assessed the effect of PKM2 knockdown on the cell’s response to doxorubicin and cisplatin, two commonly used chemotherapeutic drugs in TACE. IC_50_ values were calculated in hypoxia for each cell line treated with or without shPKM2 knockdown and scramble control. In all cell lines, we noted a reduction in the hypoxia IC_50_ of these two chemotherapeutics (Fig. 4a-b). Among responder-like cells treated with shPKM2, Hep3B demonstrated a 0.56 (*p* < 0.01) and 0.41 (*p* < 0.01) fold change in the IC_50_ of doxorubicin and cisplatin compared to scramble control, respectively, whereas HUH7 demonstrated a 0.32 (*p* < 0.01) and 0.31 (*p* < 0.01) fold change in the IC_50_ of doxorubicin and cisplatin. In addition, non-responder like cells treated with shPKM2, SNU-387 demonstrated a 0.13 (*p* < 0.01) and 0.18 (*p* < 0.01) fold change in the IC_50_ of doxorubicin and cisplatin while SNU-475 demonstrated a 0.07 (*p* < 0.01) and 0.08 (*p* < 0.01) fold change in the IC_50_ of doxorubicin and cisplatin respectively in hypoxia.

Next, we designed an in vitro TACE simulation assay to capture the effect of both chemotherapy and acute hypoxia in which cells were treated at their IC_50_ of doxorubicin and placed into a closed 1% oxygen system to induce acute hypoxia. Given that doxorubicin is the most commonly used TACE agent worldwide, and its slightly larger reduction in IC_50_ in PKM2 knockdown compared to cisplatin, we elected to perform further characterization of in vitro TACE model using doxorubicin. Cellular proliferation was then measured over 72 h. As expected, the responder cell lines had a significant response to doxorubicin alone (Fold change: Hep3B: 0.22, *p* < 0.01; HUH7: 0.01, *p* < 0.01), which was slightly improved when cells were treated with shPKM2 (Fold change: Hep3B: 0.09, *p* < 0.01; HUH7: 0.05, *p* < 0.01) (Fig. 4c Left panels). Conversely, amongst the non-responder cell lines, SNU387 had no response to doxorubicin (Fold Change:1.24 *p* = 0.33) whereas SNU475 had a minimal response (Fold Change: 0.87, *p* < 0.01) (Fig. 4c, right panels). When non-responder cells were treated with shPKM2 and doxorubicin, both non-responder cell lines had a drastic reduction of cell growth (Fold Change: SNU-387: 0.03, *p* < 0.01); SNU-475: 0.12, *p* < 0.01) (Fig. 4c, Right panels). These data suggest that PKM2 inhibition was able to inhibit cell growth in all cell lines, resulting in improved TACE-like efficacy in vitro (Fig. 4d).

### Pharmacologic inhibition of PKM2 sensitizes non-responder cells to doxorubicin and improves TACE response in vitro

**Fig. 5:**
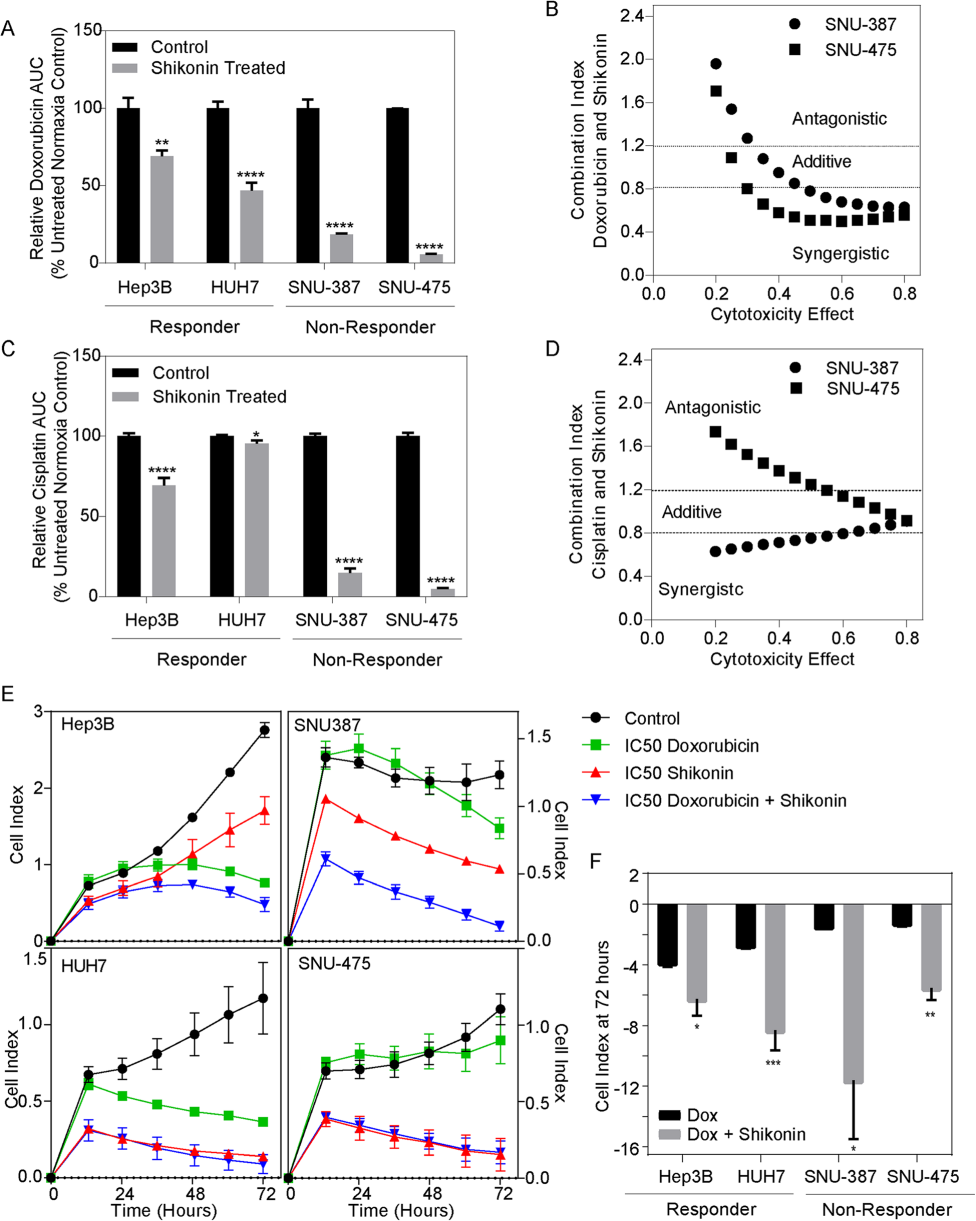
Shikonin Sensitizes Cells to Chemotherapy and Improves TACE Response In Vitro: **(a)** Relative decrease in the doxorubicin and cisplatin area under the curve when cells are treated concomitantly with shikonin in hypoxia when treated at two fold dilutions at starting concentration of 10 μM to control **(b)** Combination index of non-responder like cells at multiple cytotoxic effects demonstrating a trend towards a synergistic effect with a doxorubicin to shikonin ratio of 1:1 whereas cisplatin and shikonin demonstrate an additive effect at a ratio of 4:1 **(c)** Relative decrease in the cisplatin area under the curve when cells are treated concomitantly with shikonin in hypoxia when treated at two fold dilutions at starting concentration of 40 μM **(d)** Combination index of non-responder like cells at multiple cytotoxic effects demonstrating a trend towards an additive effect with a cisplatin to shikonin ratio of 4:1 **(e)** In vitro TACE assay measuring cellular proliferation at 72 h in hypoxia after the administration of doxorubicin at cell specific IC_50_ with the addition of shikonin at cell specific IC_50_**(f)** Relative change in cellular proliferation demonstrating therapeutic reprogramming in TACE non-responders with the addition of shikonin

Shikonin, a naphthoquinone, has been shown to inhibit glycolysis through PKM2 specific inhibition [17]. We next sought to evaluate whether shikonin had similar inhibitory effects in our TACE response cell models. We first tested the effect of shikonin on the sensitization of cancer cells to either doxorubicin (Fig. 5a) or cisplatin (Fig. 5c). In the responder-like cell lines, Hep3B demonstrated a reduction in the area under the curve (AUC) in hypoxia of 31% (*p* < 0.01) with doxorubicin and 31% (*p* < 0.01) with cisplatin whereas HUH7 demonstrated a 53% reduction (*p* < 0.01) and a 5% reduction (*p* = 0.02) with doxorubicin and cisplatin, respectively. The non-responder-like cell lines also experienced a decrease in the AUC with combination chemotherapy and shikonin. SNU-387 demonstrated an 82% (*p* < 0.01) and 85% (*p* < 0.01) reduction in AUC with doxorubicin and cisplatin, respectively, whereas SNU-475 demonstrated a 94% (*p* < 0.01) reduction in AUC with doxorubicin and a 95% (*p* < 0.01) AUC reduction with cisplatin. To determine the interaction between both doxorubicin and shikonin and cisplatin and shikonin in the non-responder like cell lines, we calculated the combination index via the Chou-Talalay method (Fig. 5b, d). We observed that doxorubicin and shikonin are synergistic at higher level of cytotoxicity (Fig. 5b), whereas the combination of shikonin and cisplatin interact in an additive manner (Fig. 5d). These data indicate that the combination therapy may be a viable option to improve TACE.

Next, we performed in vitro TACE simulation with the addition of shikonin and doxorubicin. Consistent with the above observations, responder-like cells had a significant decrease in cellular proliferation with doxorubicin alone (Fold change: Hep3B: 0.27, *p* < 0.01; HUH7: 0.31, *p* < 0.01), which was further enhanced with the combination therapy of shikonin and doxorubicin (Fold change: Hep3B: 0.15, *p* < 0.01; HUH7:0.10, *p* < 0.01) (Fig. 5e, Left panels). Conversely, doxorubicin alone had a small reduction in cellular proliferation in both non-responder like cell lines (Fold change: SNU-387: 0.64, *p* < 0.01; SNU-475: 0.81, *p* = 0.04) but with the addition of shikonin therapeutic efficacy was significantly increased which resulted in a 0.11 (*p* < 0.01) fold change in the SNU-387 cells and a 0.13 (*p* < 0.01) fold change in the SNU-475 cells (Fig. 5e, Right panels). These data suggest that resistance can be overcome with the addition of a PKM2 inhibitor during the TACE procedure, which has the potential to greatly improve treatment response. Additionally, we observed that combination therapy with doxorubicin and shikonin significantly improved in vitro TACE response in all cell lines (Fig. 5f). This data may suggest that combination shikonin and doxorubicin has the potential to improve TACE efficacy even in those patients with expected favorable outcomes.

### Patient derived non-responder like organoids can undergo therapeutic reprogramming

**Fig. 6:**
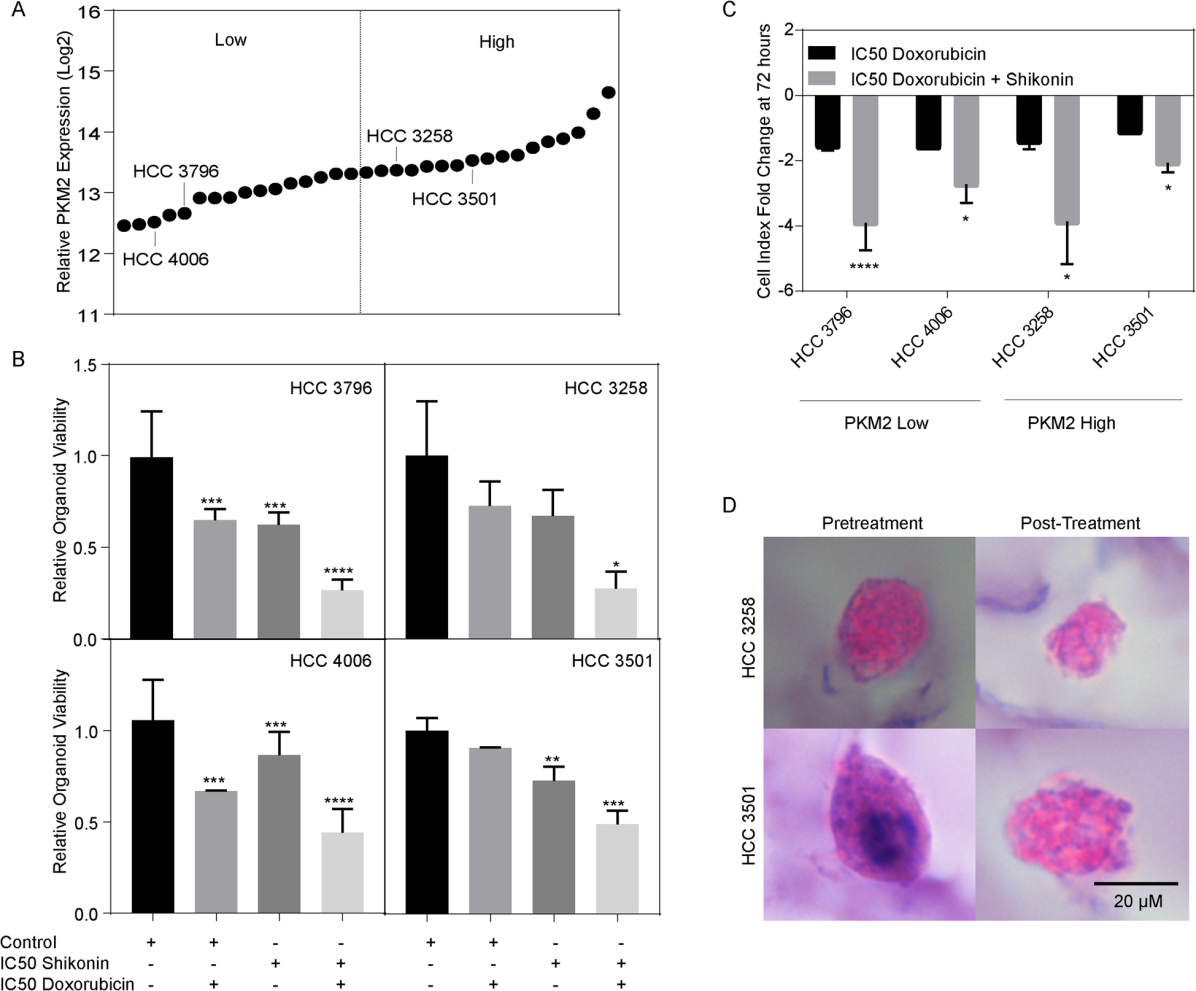
Combination shikonin and doxorubicin reprograms TACE response in patient derived organoids. **(a)** 33 HCC patient derived cells lines were screened for relative mRNA expression of PKM2. Two representative cell lines were selected from PKM2 high lines and two representative PKM2 low lines for downstream analysis. **(b)** Organoid viability was measured after 72 h of treatment with doxorubicin with or without shikonin in addition to subjection of acute hypoxia. PKM2 low lines exhibited decreased viability with doxorubicin alone whereas PKM2 high lines demonstrated resistance to doxorubicin which could be overcome with the combination of doxorubicin and shikonin **(c)** Relative change in doxorubicin and shikonin response as compared to doxorubicin alone demonstrating decreased viability with the combination of shikonin and doxorubicin in all line lines. **(d)** Histology of patient derived organoid before treatment and after combination doxorubicin and shikonin treatment in hypoxia

To test the hypothesis that combination shikonin and doxorubicin improves TACE response in pre-clinical models*,* we used a 3D model utilizing PDCs to better approximate a tumor-like environment. Given our findings that PKM2 enrichment both confers a negative prognostic outcome as well as is associated with TACE resistance, we elected to screen 33 HCC PDCs for PKM2 expression. Utilizing the NanoString platform, median relative PKM2 mRNA expression was 13.33 (Fig. 6a). We then selected two representative lines from both the PKM2-high and PKM2-low groups and generated 3D organoids. In a similar manner to our 2D TACE in vitro assay, organoids were treated at cell line specific IC_50_s of both shikonin and doxorubicin and subjected to hypoxia. Organoid viability was then assessed. PKM2 low cells behaved in a similar manner to the responder-like cells in 2D culture with both cell lines demonstrating a statistically significant decrease in viability upon doxorubicin treatment alone (Viability fold change: HCC 3796: 0.64, *p* < 0.01; HCC 4006: 0.67, *p* = 0.03) (Fig. 6b, Left panels). Conversely, PKM2 high cells behaved in a similar manner when compared to the TACE non-responder cells. Both HCC 3258 and HCC 3501 demonstrated resistance to doxorubicin treatment alone (Viability fold change: HCC 3258: 0.74, *p* = 0.37; HCC 3501: 0.91, *p* = 0.34) whereas when treated with the combination of doxorubicin and shikonin, resistance is overcome resulting in a 0.27 (*p* = 0.01) and 0.49 (*p* < 0.01) fold change in HCC 3258 and HCC 3501 respectively (Fig. 6b, Right panels). In addition to the improved response in non-responders, we also noted a significant difference in the response to doxorubicin and combination treatment regardless of PKM2 expression (Fig. 6c). Finally, we examined the histologic characteristics of the PKM2 High organoids both before and after combination therapy. In a pretreatment condition, HCC spheres displayed a well circumscribed structure whereas following combination treatment they appeared more disorganized and irregular. Similarly, the results seen in our 2D model, we observed increased efficacy of our in vitro TACE assay when non-responder like organoids were treated with combination PKM2 inhibitor and standard chemotherapy.

## Discussion

Unlike many other solid organ tumors where advances in detection and treatment have resulted in improved survival, HCC deaths among both men and woman continues to rise [3, 18]. In patients with advanced disease, the only option was Sorafenib, a multi tyrosine kinase inhibitor, which in the landmark SHARP trial conveyed a less than three-month survival advantage compared to supportive care [19]. The recent introduction of nivolumab and regorafenib in the second line and lenvatinib in the first line offer only modest benefit [20–22]. Where systemic therapy has failed, locoregional therapies such as TACE for intermediate HCC have had some success. As previously stated, while benefit can be seen, patient selection is paramount, with more than 40% of patient having no response to therapy. In addition, the definition of intermediate HCC comprises only 10–12% of patients at the time of diagnosis, further limiting treatment [23]. This highlights the need for both improvement in the efficacy of TACE as well as improved methods of patients’ selection to better identify who will benefit from treatment. Our approach to improve TACE efficacy is focused on overcoming the mechanism of resistance. We demonstrate that through manipulation of cellular biology we are able to greatly increase chemotherapeutic sensitivity.

Since first described in the 1920’s by Otto Warburg, the propensity for cancer cells to preferentially utilize glycolysis even in the presence of oxygen has been well documented [24, 25]. While still a topic of much debate, the notion that cancer cells undergo metabolic reprogramming has been dubbed an “emerging hallmark” of cancer [26]. From our data we demonstrate that glucose metabolism dysregulation is heterogenous amongst different patient populations. We also demonstrate that the degree of glycolysis dysregulation is associated with TACE resistance. PKM2 represents the more common of the two splice variants of PKM gene and is abundant in tumors [16]. PKM2 expression has been shown to be elevated in many cancer types and specifically in HCC has be shown to portray a negative prognosis [27]. Our findings show that within the specific subset of patients treated with TACE, elevated PKM2 expression is associated with attenuated survival. In addition, we have demonstrated experimentally that increased expression of PKM2 is associated with increased glycolysis activity. With the knowledge that glycolysis enrichment is related to TACE resistance and PKM2 expression is associated with glycolysis activity, these findings further support the role of PKM2 as a contributor to TACE resistance. In addition, we have observed that all cell lines experience a change in cancer associated phenotypes when PKM2 was knocked down, indicating that this gene may be a suitable candidate to target. In fact, in SNU-387 cells, PKM2 knockdown revealed a relative change in IC_50_ of both doxorubicin and cisplatin that was more dramatic than either of the responder-like cell lines. A possible explanation for this response may be related to the relative expression of PKM2 in responder-like compared to non-responder-like cell lines, allowing for a differential effect with PKM2 knockdown, however, further mechanisms remain be elucidated.

It has been well established that traditional systemic chemotherapy is ineffective in HCC [28]. Conversely, when administered directly into hepatic arterial circulation, it does appear to convey a survival advantage as compared to embolization alone [23]. Therefore, there is a need to improve the response to TACE chemotherapy, as it appears that hypoxia alone does not induce a sufficient enough tumor response to result in prolonged survival. In the United States, TACE is most commonly performed with a single agent chemotherapy, with doxorubicin and cisplatin representing the two most common agents [5]. Current efforts to improve TACE have focused on the reformulation of doxorubicin as a drug eluting bead (DEB-TACE) with the hypothesis that a more controlled doxorubicin release would improve response rates. While superior to bland embolization, the Precision V trial of DEB-TACE to conventional TACE failed to show improved response [29, 30]. Here, we have demonstrated that PKM2 inhibition resulted in improved doxorubicin and cisplatin sensitivity in HCC cell lines. In addition, with an in vitro TACE assay we demonstrated that previously resistant cell lines become sensitized to therapy. Therefore, we believe that the inhibition of PKM2 inhibition may not only improve response rate but may also improve the magnitude of response in select patients.

As with any proposed targeted therapy, a drug should ideally be specific, minimizing off-target effects while maintaining an acceptable burden of adverse events. While RNA interference has proven to be a successful tool in identification and validation of potential lethal targets in cancer research, its application in clinical practice is limited [31, 32]. We therefore set out to demonstrate that a pharmacologic inhibitor of PKM2 could produce similar results. Currently there are three available PKM2 inhibitors, the small molecule Compound 3, a naphthoquinone, shikonin and its analog alkannin [16]. While shikonin’s anti-cancer properties has been studied for some time, Chen et al. identified its mechanism as an inhibitor of glycolysis by targeting PKM2 [17]. Additionally, shikonin and its analog were observed to have a much greater effect on PKM2 activity than Compound 3. We demonstrate that doxorubicin in combination with shikonin had a greater effect on the proliferation of cell lines and viability of PDC organoids than either treatment alone when used together in a TACE simulation and thus this combination represents a reasonable therapeutic approach to test in clinical trials. Lastly, given that we opted to characterize our in vitro TACE model with doxorubicin, it is possible that using different chemotherapy agents will produce differential effects in combination with PKM2 inhibition. Future work will aim at further characterizing this model using a larger selection of chemotherapy agents, as well as combination therapy to identify optimal agents for combination with PKM2 inhibition.

## Conclusions

We demonstrate a potential strategy by which the TACE procedure can be augmented through targeted therapeutic reprogramming through the inhibition of PKM2. We have established that enrichment of PKM2 is a poor prognostic indicator and represents a pivotal role in the altered glucose metabolism observed in non-responders. Furthermore, we have demonstrated that the inhibition of PKM2 results in decreased cell viability and improved response to commonly used TACE chemotherapeutics. More importantly, we demonstrated that when PKM2 is inhibited, treatment resistance can be overcome, and cell death can be induced. Finally, we showed that with a known pharmacologic inhibitor, shikonin, TACE efficacy in vitro is greatly improved. We believe that these findings lay the groundwork for future clinical trials, with the potential to improve patient outcomes.

## Supplementary information

**Additional file 1:Table S1.** IC_50_ Concentrations Used For Invitro Experiments. **Table S2.** Univariable and Multivariable Cox Proportional Hazards Model of LCI cohort. **Figure S1.** Gene expression and survival analysis of gluconeogenesis related genes correlated with TACE navigator. **Figure S2.** Gene expression and survival analysis of PKM2 in Hong Kong cohort. **Figure S3.** Dose effect curves for patient derived cell lines.

## Data Availability

The datasets used and/or analysed during the current study are available from the corresponding author on reasonable request.

## Abbreviations

HCC: Hepatocellular carcinoma
BCLC: Barcelona clinic liver cancer stage
TACE: Transarterial chemoembolization
PDC(s): Patient derived cell line(s)
PKM2: Pyruvate kinase muscle isoenzyme M2
LCI: Liver cancer institute
CCLE: Cancer cell line encyclopedia
FBS: Fetal bovine serum
qRT-PCR: quantitative rea-time transcription-polymerase chain reaction
G6PC: Glucose-6-Phosphatase catalytic subunit
PCK1: Phosphoenolpyruvate carboxykinase 1
AUC: Area under the curve
DEB-TACE: Drug eluting bead TACE
