# Supplementary material for: PKM2 inhibition may reverse therapeutic resistance to transarterial chemoembolization in hepatocellular carcinoma
Source: J Exp Clin Cancer Res. 2020 Jun 3;39:99. doi: 10.1186/s13046-020-01605-y (PMC7268641; doi:10.1186/s13046-020-01605-y)

Supplemental Table 1: IC50 Concentrations Used For Invitro Experiments

|  | **Doxorubicin (µM)** | **Cisplatin (µM)** | **Shikonin (µM)** |
| --- | --- | --- | --- |
| **Hep3B** | 0.0364 | 14.2 | 0.276 |
| **HUH7** | 0.261 | 15.1 | 1.13 |
| **SNU-387** | 0.236 | 18.2 | 0.858 |
| **SNU-475** | 1.51 | 19.0 | 9.51 |
| **HCC 3501** | 2.27 | - | 1.4 |
| **HCC 3796** | 1.09 | - | 6.0 |
| **HCC 4006** | 0.63 | - | 4.18 |
| **HCC 3258** | 4.1 | - | 1.8 |

Supplemental Table 2: Univariable and Multivariable Cox Proportional Hazards Model of LCI cohort

|  | **Univariable** | | | **Multivariable** | | |
| --- | --- | --- | --- | --- | --- | --- |
|  | **Hazard Ratio** | **95% Confidence Interval** | **p-value** | **Hazard Ratio** | **95% Confidence Interval** | **p-value** |
| PKM2 High | 3.83 | 1.87-6.93 | <0.01 | 3.02 | 1.50-6.04 | <0.01 |
| Age | 0.98 | 0.95-1.01 | 0.27 | - | - |  |
| Female Sex | 0.78 | 0.24-2.53 | 0.68 | - | - |  |
| Child B Score | 1.28 | 0.48-3.42 | 0.62 | - | - |  |
| BCLC Stage B | 1.41 | 0.49-4.06 | 0.52 | 0.95 | 0.33-2.76 | 0.93 |
| BCLC Stage C | 3.94 | 1.97-7.59 | <0.01 | 2.62 | 1.30-5.27 | <0.01 |
| Satellite Nodules | 0.75 | 0.28-2.20 | 0.65 | - | - |  |
| Multiple Nodules | 1.19 | 0.59-2.42 | 0.63 | - | - |  |
| Encapsulated Tumor | 0.40 | 0.58-2.27 | 0.39 | - | - |  |
| Local Invasion | 3.48 | 1.45-3.38 | <0.01 | 1.92 | 0.78-4.73 | 0.15 |
| Cirrhosis | 7.81 | 1.07-56.82 | 0.04 | 5.81 | 0.79-42.71 | 0.08 |
| AFP>300 ng/ml | 1.58 | 0.84-2.98 | 0.16 | - | - |  |

Supplemental Figure 1: Gene expression and survival analysis of gluconeogenesis related genes correlated with TACE navigator


Supplemental Figure 2: Gene expression and survival analysis of PKM2 in Hong Kong cohort

Supplemental Figure 3: Dose effect curves for patient derived cell lines.


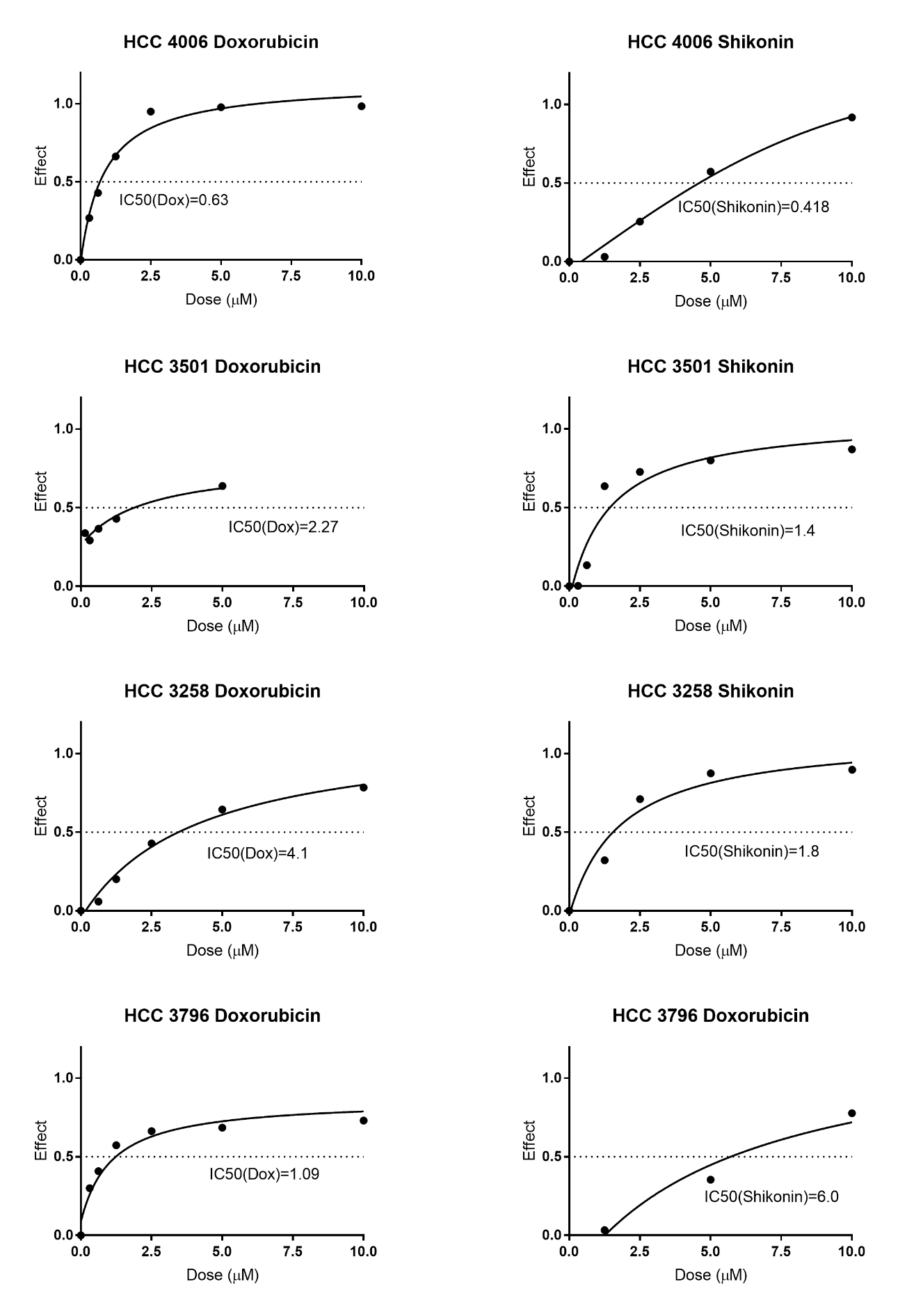

Supplement: Supplementary file 1 — Additional file 1:Table S1. IC50 Concentrations Used For Invitro Experiments. Table S2. Univariable and Multivariable Cox Proportional Hazards Model of LCI cohort. Figure S1. Gene expression and survival analysis of gluconeogenesis related genes correlated with TACE navigator. Figure S2. Gene expression and survival analysis of PKM2 in Hong Kong cohort. Figure S3. Dose effect curves for patient derived cell lines. [file 13046_2020_1605_MOESM1_ESM.docx]
